# Relevance of arginine residues in Cu(II)-induced DNA breakage and Proteinase K resistance of H1 histones

**DOI:** 10.1038/s41598-018-25784-z

**Published:** 2018-05-09

**Authors:** Marina Piscopo, Marco Trifuoggi, Carmela Scarano, Carla Gori, Antonella Giarra, Ferdinando Febbraio

**Affiliations:** 1 Dipartimento di Biologia, Università degli Studi di Napoli Federico II, 80126 Napoli, Italy; 2 Dipartimento di Scienze Chimiche, Università degli Studi di Napoli Federico II, 80126 Napoli, Italy; 3 CNR, Institute of Protein Biochemistry, 80131 Napoli, Italy

## Abstract

This work analyzes the involvement of arginines in copper/H_2_O_2_-induced DNA breakage. Copper is a highly redox active metal which has been demonstrated to form compounds with arginines. For this aim we used mixtures of pGEM3 DNA plasmid and two types of H1 histones which differ only in their arginine content. The sperm H1 histone from the annelid worm *Chaetopterus variopedatus* (arginine content 12.6 mol% K/R ratio 2) and the somatic H1 histone from calf thymus (arginine content 1.8 mol% and K/R ratio 15). Copper/H_2_O_2_-induced DNA breakage was observed only in presence of sperm H1 histones, but it was more relevant for the native molecule than for the deguanidinated derivative (K/R ratio 14), in which 80% of arginine residues were converted to ornithine. Further, copper induced proteinase K resistance and increase of DNA binding affinity on native sperm H1 histones. These observations are consistent with a copper induced reorganization of the side-chains of arginine residues. Copper, instead, did not affect DNA binding affinity of somatic and deguanidinated H1 histones, which show similar K/R ratio and DNA binding mode. These results indicate that arginine residues could affect these H1 histones properties and provide new insights into copper toxicity mechanisms.

## Introduction

Copper ions play important roles in many chemical and biochemical processes and are required for cellular respiration, peptide amidation, neurotransmitter biosynthesis, pigment formation and connective tissue strength^1^. In many cases, the functions in these processes result from copper ions either as mono-metal or multi-metal complexes of peptides or proteins^2–5^. Copper works also as cofactor for numerous enzymes and plays an important role in central nervous system development; low concentrations of copper may result in incomplete development, whereas excess copper maybe injurious, in fact copper is considered one of the strongly suspected etiological factors in some neurodegenerative disorders^6^. Many studies indicated that copper-overload readily leads to oxidative stress, indeed most of the toxicity of oxygen and hydrogen peroxide *in vivo* arises from metal ion-catalyzed production of highly reactive hydroxyl radical (^**·**^OH) by the Fenton reaction, which is illustrated as follows:

1 $${\rm{Fe}}({\rm{II}})/\mathrm{Cu}({\rm{I}})+{{\rm{H}}}_{{\rm{2}}}{{\rm{O}}}_{{\rm{2}}}\to {\rm{Fe}}({\rm{III}})/\mathrm{Cu}({\rm{II}})+{}^{\cdot }{\rm{OH}}+{{\rm{OH}}}^{-}$$

The hydroxyl radical is extremely reactive and can further react with practically any biological molecules in the near vicinity, causing catastrophic damages to lipids, proteins and DNA^7^ and is critically involved in copper cytotoxicity^8–12^. Also Cu(II) ions can participate in formation of ^**·**^OH through the Fenton reaction and appear to be potentially more reactive in mediating oxygen radical-induced cytotoxicity and genotoxicity than iron ions^13^. Moreover, copper can induce oxidative stress also by significantly decreasing of glutathione levels^14^.

In addition, copper being a non-degradable heavy metal, can accumulate in soil or leach into water sources. Its accumulation has impacted micro and macro organisms^15,16^; expecially marine organisms^17,18^ spurring scientists to research *in situ* copper removal methods^15^.

It has been previously reported^19^ that DNA damage in the presence of copper and H_2_O_2_ occurs in a multistage mechanism in which, firstly Cu(II) but not Cu(I) binds to an electronegative region involving at least two guanosines. As a second step Cu(II) reacts with DNA, perhaps through proton transfer involving guanine. This reduction of Cu(II) could yield oxidation products of guanine which are piperidine sensitive. In the last step H_2_O_2_ reacts with the Cu(I) formed, either still bound or in the proximity of DNA, generating ^**·**^OH and regenerating Cu(II). In turn, ^**·**^OH produces (additional) piperidine- sensitive base damage and/or strand breaks at short range from the original Cu(II)-binding site. Furthermore, it has also been reported^20^ that a class of binuclear and trinuclear copper complexes show high selectivity in oxidizing DNA at ss/ds DNA junctions, but an efficient cleavage was not observed for ss or ds DNA alone, and that also the flexibility of the DNA strand is an important factor in the ss/ds junction selectivity. In living cells, DNA is not free but complexed with histones to form chromatin^21^. Although histone proteins are known to protect DNA from a variety of potentially dangerous reactive species, such as hydroxyl radicals (^**·**^OH), the packaging within the nucleosome does not protect DNA from metal ion-dependent free radical damage^22^. Since DNA is blocked by the physiological cation Mg^2+^, copper is likely to associate predominantly with histones, which may also react with free radicals. DNA bases may participate in the formation of DNA-protein cross-links in chromatin^21^ and electron transfer easily occurs from the histone to DNA, leading to DNA damage^23^.

In particular, it seems that some interactions of DNA with peptides can increase metal/H_2_O_2_ induced DNA breakage^24^ and/or these reactions can lead to oxygen activation that in turn can proceed *in vivo* around and inside the cell nucleus^25^. For example, the peptides H2B32-62 and H2B63-93, as well as the N terminal tail (H2B1-31) of histone H2B are able to enhance copper induced single and double strand scission of plasmid DNA^26^ and H4 histone peptide of AKRHRK efficiently enhanced Cu(II)/H_2_O_2_ induced DNA damage, especially at cytosine residues^27^. As a matter of fact DNA damage induced by Cu(II)/H_2_O_2_ is enhanced in the nucleosomal compared to the isolated DNA^28^. In addition to the free radical-induced oxidative damage, information available suggests that the cellular response to copper overload, particularly at the early stages of copper accumulation, involves more specific mechanisms and pathways. In order to provide new insights in the mechanism of copper toxicity, in this work we evaluated the possible involvement of arginine residues in Cu(II)/H_2_O_2_-induced DNA breakage since in literature it has been reported the possibility of several binary and ternary copper compounds of arginine^29^. To this aim we analyzed Cu(II)/H_2_O_2_-induced DNA breakage in presence of sperm and somatic H1 histones. Sperm H1 histone was extracted from the sperm chromatin of the annelid worm *Chaetopterus variopedatus* (*Ch*.*v*.); somatic one was from calf thymus (C.T.). These H1 histones show different K/R ratios (2 and 15 respectively) because differ substantially only in their arginine content (12.6 mol % and 1.8 mol % respectively). This different arginine content affects the H1 histones DNA binding mode^30^. Furthermore, since proteins are the most abundant target within cells for radicals such as ^**·**^OH^31^ and heavily oxidized proteins generally show decreased susceptibility to proteolytic attack by most proteinases^32,33^, we studied also the relevance of arginine residues in Cu(II)-induced proteinase K resistance of these two types of H1 histones also because in literature several studies have suggested that Cu(II) ions convert some proteins to a proteinase K-resistant conformation^34,35^.

## Results

### Analysis of Cu(II)-induced effects on sperm and somatic H1 histones in DNA binding

**Figure 1:**
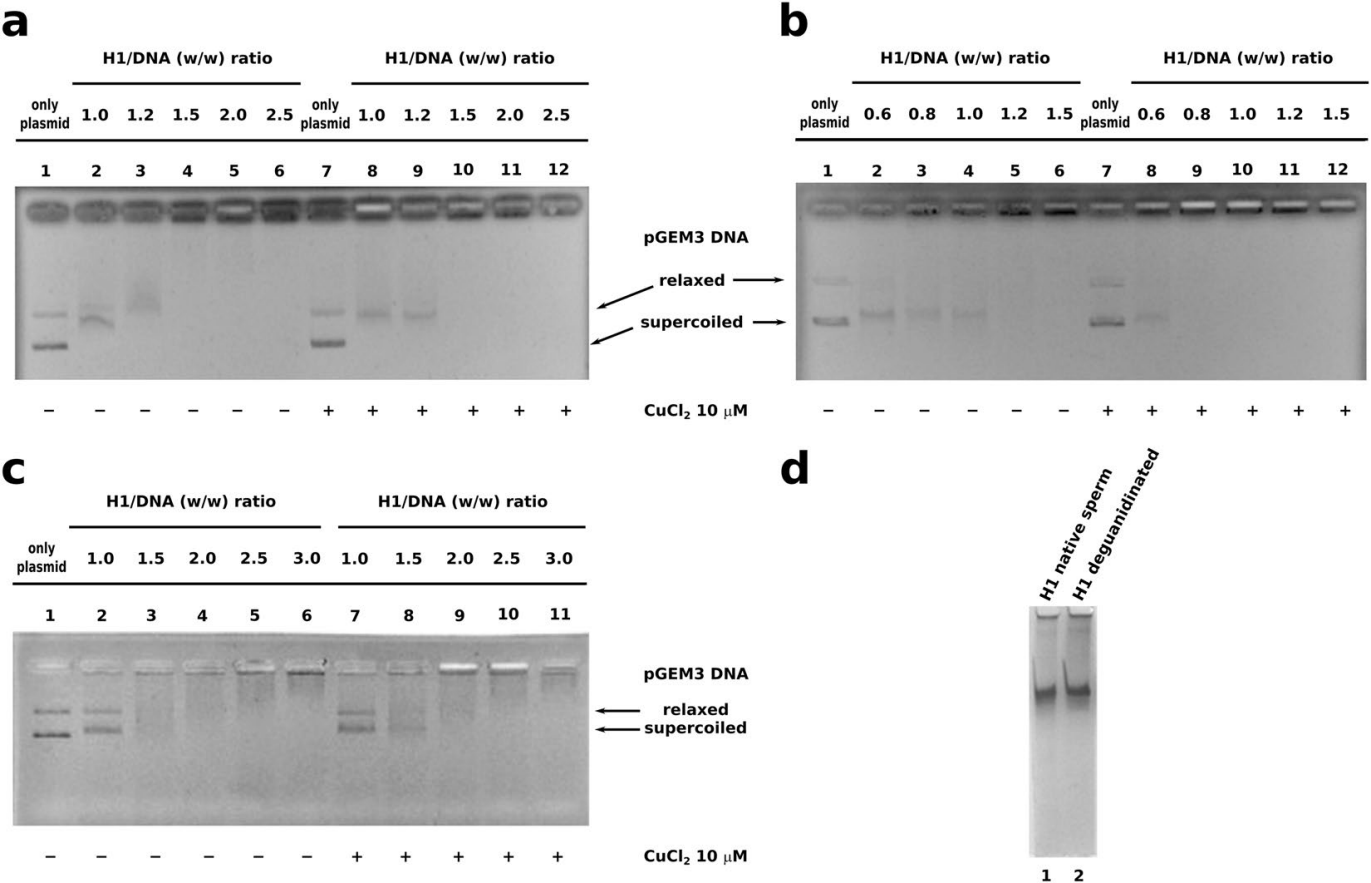
DNA binding affinity of H1 histones in absence and presence of 10 µM CuCl_2_ analized by EMSA. Samples containing pGEM3 plasmid DNA, incubated with increasing amount of (**a**) somatic H1 histones, (**b**) native sperm H1 histones and (**c**) deguanidinated sperm H1 histones, were analyzed by 1% agarose gel electrophoresis. (**d**) AU-PAGE of native and deguanidinated sperm H1 histones.

In order to evaluate the effect of CuCl_2_ on DNA binding of sperm and somatic H1 histones, we performed EMSA with these two types of H1 histones using pGEM3 plasmid DNA. The results obtained without CuCl_2_, shown in Fig. 1a (lanes 1–6) and Fig. 1b (lanes 1–6) are in agreement with those reported in Piscopo *et al*.^26^ regarding the different DNA binding modes that we defined: “all or nothing” and “intermediate” mode for sperm and somatic H1 histones respectively. EMSA performed with pGEM3 DNA plasmid somatic and sperm and H1 histones in presence of 10 µM CuCl_2_ (Fig. 1a, lanes 7–12; Fig. 1b, lanes 7–12) showed that this salt, at this concentration, didn’t change the DNA binding mode of both H1 histones but produced only on native sperm H1 histone an increase of DNA binding affinity. In fact, in presence of 10 µM CuCl_2,_ we observed the formation of the single DNA band with low mobility, close to the well^30^, already at H1/DNA (w/w) ratio 0.8 instead of 1.2 (Fig. 1b, lanes 9 and 5), while in the case of somatic H1 histones the results obtained with or without CuCl_2_ were very similar (Fig. 1a). The same analysis performed on deguanidinated derivatives of sperm H1 histones, in which 80% of arginine were converted in ornithine residues (K/R = 14), showed a DNA binding mode more similar to that of somatic H1 histones and not influenced by CuCl_2_ (Fig. 1c). Moreover, we observed a lower DNA binding affinity of deguanidinated derivatives with respect to native molecules, as shown in Fig. 1c because the single DNA band with low mobility, close to the well, was not achieved even at H1/DNA (w/w) ratio 3. The decrease of DNA binding affinity of deguanidinated sperm H1 histones could depend only on the conversion of arginine in ornithine residues because as shown in the AU-PAGE of Fig. 1d (lane 2), the molecule, after deguanidination, appears not degradated and with similar mobility with respect to native one (Fig. 1d, lane 1).

### Cu(II)/H_2_O_2_-induced DNA breakage in presence of H1 histones

**Figure 2:**
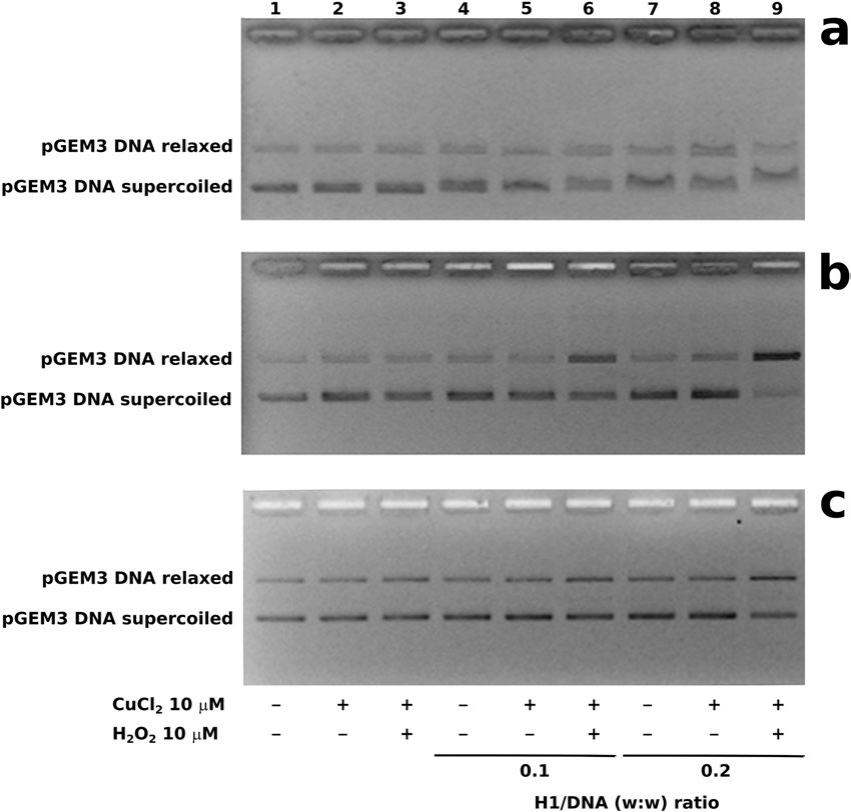
Analysis by electrophoresis in 1% agarose gel of pGEM3 plamid DNA breakage induced by Cu(II)/H_2_O_2_ in presence of (**a**) somatic H1 histones, (**b**) native sperm H1 histones and (**c**) deguanidinated sperm H1 histones.

In Fig. 2, the results of the analyses of Cu(II)/H_2_O_2_-induced DNA breakage in presence of H1 histones, are shown. The analyses were performed using pGEM3 DNA plasmid and native and deguanidinated sperm H1 histones and somatic ones, in presence of 10 μM CuCl_2_ and 10 μM H_2_O_2_. DNA breakage was evaluated by the conversion of supercoiled to relaxed form of plasmid DNA. In our conditions, DNA breakage is not observed when plasmid is mixed with CuCl_2_ in absence or presence of H_2_O_2_ (Fig. 2a–c, lanes 2–3), being necessary higher H_2_O_2_ concentration, at least 100 µM in order to cause DNA breakage (Supplementary Figure S1). Similarly, the absence of a DNA breakage is also observed using H1 histones in presence or absence of CuCl_2_ (Fig. 2a–c, lanes 4–5, 7–8). The addition of CuCl_2_ to a mixture of native sperm H1 histones/DNA in a 0.1 (w/w) ratio in presence of H_2_O_2_, determines an increase of the amount of relaxed plasmid DNA form at detriment of the supercoiled one (Fig. 2b, lane 6). This effect resulted more relevant with sperm H1 histone/DNA 0.2 (w/w) ratio, because in this case, plasmid DNA appears almost completely in the relaxed form (Fig. 2b, lane 9). The same analysis performed with somatic H1 histones didn’t show DNA breakage in none of the conditions but only a reduction of plasmid DNA supercoiling degree at H1 histone/DNA 0.2 (w/w) ratio in presence of H_2_O_2_ and CuCl_2_ (Fig. 2a, lane 9). Deguanidinated sperm H1 histone derivatives instead were still able to induce DNA breakage but with lesser efficiency respect to native molecules at H1 histone/DNA 0.1 and 0.2 (w/w) ratio in presence of H_2_O_2_ and CuCl_2_ (Fig. 2c, lanes 6 and 9).

### H1 histones resistance to PK digestion

**Figure 3:**
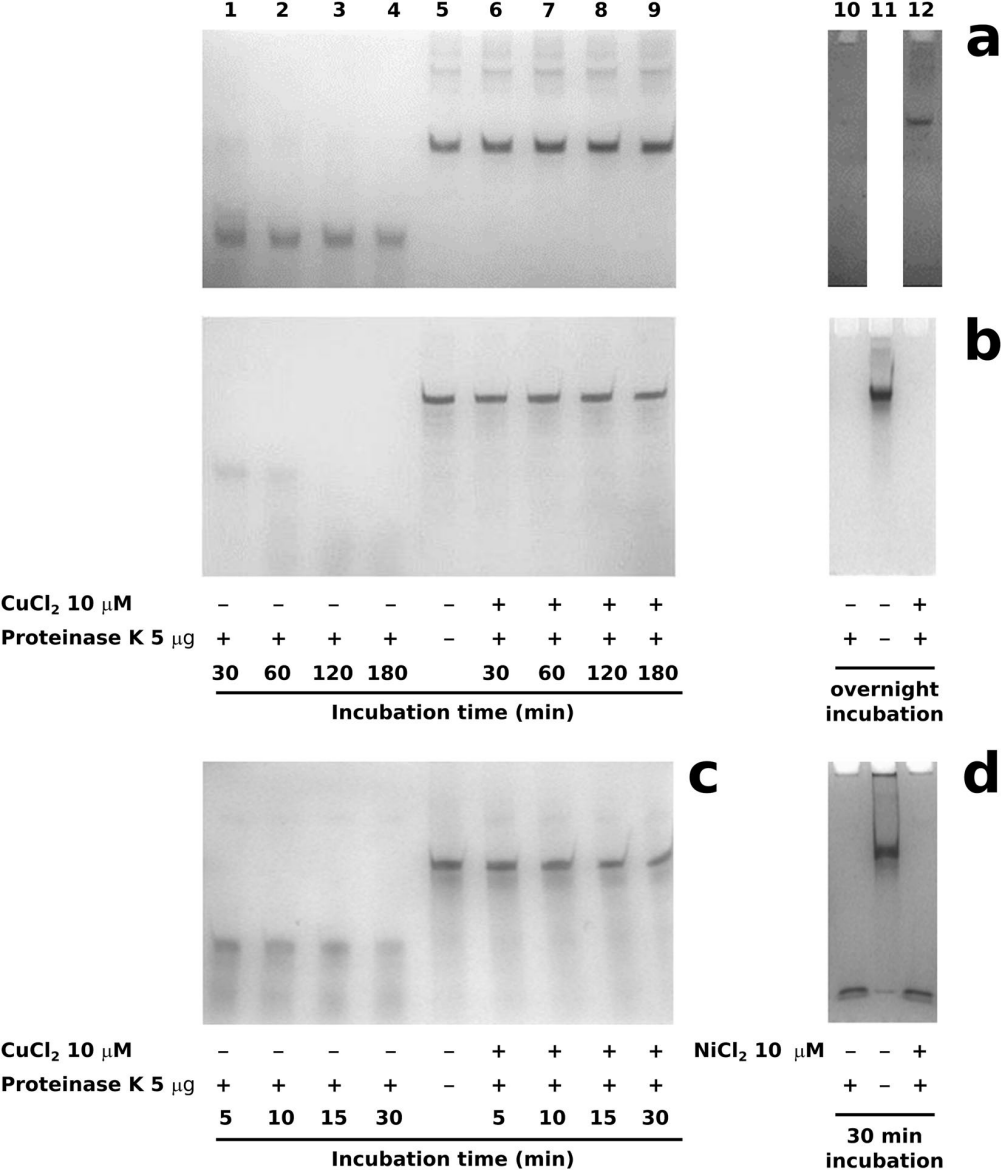
Proteinase K digestion kinetics of H1 histones in absence and presence of 10 μM CuCl_2_ analized by AU-PAGE. (**a**) Native sperm H1 histones digested for 30′-3 h and overnight; (**b**) Somatic H1 histones digested for 30′-3 h and overnight; (**c**) Deguanidinated sperm H1 histones digested for 5′–30′; (**d**) Native sperm H1 histones digested for 30′ in absence and presence of 10 μM NiCl_2_.

In Fig. 3, the AU-PAGE of the results of PK digestions of native and deguanidinated sperm H1 histones and somatic ones in presence of 10 µM CuCl_2_ are shown. Native sperm H1 histones, (Fig. 3a, lanes 1–4) showed higher PK resistance compared with the somatic ones (Fig. 3b, lanes 1–4), since complete digestion of native sperm H1 histones was obtained only after an overnight PK treatment (Fig. 3a, lane 10), while somatic H1 histones were completely digested, (Fig. 3b, lanes 1–4), already within 3 h of PK treatment. In presence of 10 µM CuCl_2_, PK digestion of native sperm H1 histones was completely inhibited (Fig. 3a, lanes 6–9), even with an overnight PK treatment (Fig. 3a, lane 12). In presence of CuCl_2_, also somatic H1 histones acquired PK resistance (Fig. 3b, lanes 6–9), but were completely digested after an overnight treatment with PK (Fig. 3b, lanes 10 and 12). On the other hand, the deguanidinated sperm H1 histone derivatives resulted more susceptible to PK digestion than native molecules regardless of the presence of CuCl_2_. In fact a progressive degradation of these molecules, in short times (30 minutes) was observed also in presence of CuCl_2_ (Fig. 3c lanes 6–9) while without CuCl_2_ these molecules were completely digested within 30′ (Fig. 3c lanes 1–4). In order to exclude the possibility of a dependence from Cl^−^ of H1 histones resistance to PK digestion, we performed experiments in presence of 10 µM NiCl_2_. The results obtained on native sperm H1 histones clearly indicated that PK resistance depends just on Cu(II) because the histones were digested regardless of the presence of NiCl_2_ already after 30′ (Fig. 3d, lanes 10 and 12).

### Cu(II) induced effects on H1 histones secondary structure

**Figure 4:**
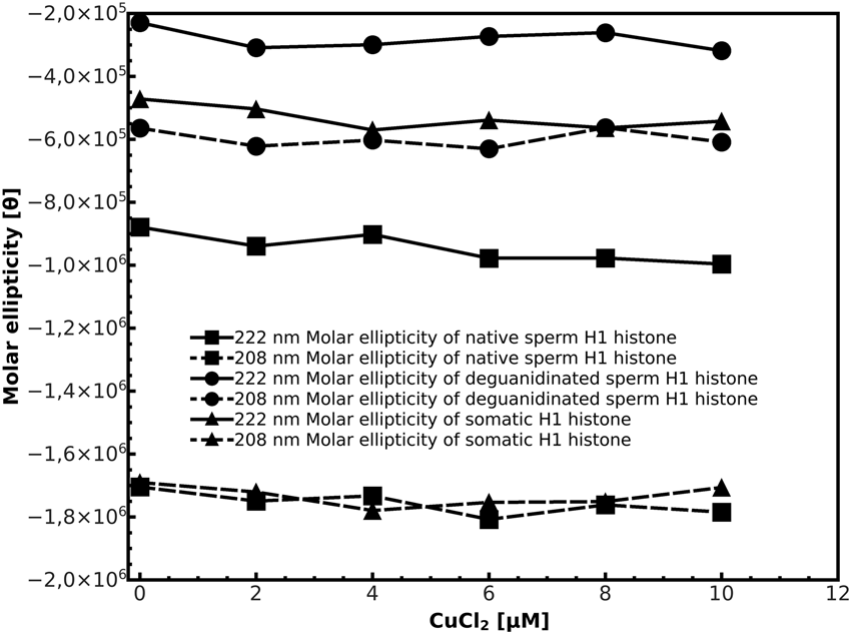
Plot of molar ellipticity values of H1 histones at the wavelengths of 208 (dashed line) and 222 nm (continued line) versus CuCl_2_ concentration in the range from 0 to 10 μM.

In order to explore the effects of Cu(II) on the structure of sperm and somatic H1 histones, we used Far-UV CD measurements, a useful tool to investigate the changes in protein secondary structure. CD spectra of H1 histones, measured in absence and in presence of increasing Cu(II) concentrations, kept similar shape (Supplementary Figure S2), retaining the molar ellipticity values at 208 and 222 nm wavelengths, as shown in the plot of molar ellipticity versus Cu(II) concentration (Fig. 4). Differences were observed between the spectra of native sperm H1 histones and its deguanidinated derivatives, which presented a loss in the molar ellipticity values of spectra peaks, indicative of changes in the secondary structure following the conversion of arginine in ornithine residues. In fact, an estimation of the content in secondary structure highlight a reduction in alpha-helix structures for the benefit of beta-sheet structures in the deguanidinated derivatives with respect the native molecules (Supplementary Figure S3A and B). Underlining that the secondary structure estimation is not a precise measure of the amount of these structures, from the box-plot of the calculated data (Supplementary Figure S3) appeared that the addition of CuCl_2_ essentially didn’t affect the amount of alpha-helix and beta-sheet structures in H1 histones, while more evident changes were observed for turn and unordered structures, that are more flexible, less structured and susceptible to environmental rearrangement of amino acid side chains.

### Fluorescence analyses of H1 histones in presence of Cu(II)

**Figure 5:**
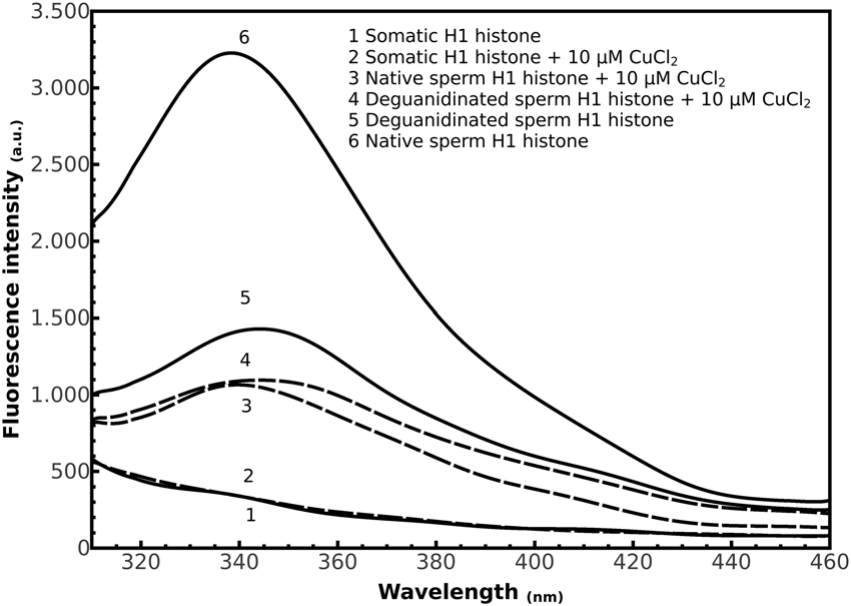
Fluorescence spectra at the excitation wavelength of 290 nm of H1 histones in absence (continued line) and presence of 10 μM CuCl_2_ (dashed line).

The measurements of intrinsic fluorescence of H1 histones were carried out following the emission signal of tyrosine residues. The absence of a strong fluorescence signal at the excitation wavelength of 290 nm of somatic H1 histones, due to the low amount of tyrosine residues in this molecule and probably to their exposition to polar environment, made impossible to measure differences in spectra of intrinsic fluorescence for this molecule in absence and in presence of Cu(II) (Fig. 5, spectra 1, 2).

The addition of CuCl_2_ resulted in a quenching of the tyrosine fluorescence either for native sperm H1 histones (Fig. 5, spectrum 3) and its deguanidinated derivatives (Fig. 5, spectrum 4) with respect their corresponding molecules without CuCl_2_ (Fig. 5, spectra 6 and 5). The Stern–Volmer plot of F_0_/F versus CuCl_2_ concentration at 15, 25 and 35 °C on native sperm H1 histones, resulted in non-linear curve, downwarding at increasing CuCl_2_ concentrations at 25 °C, and decreasing the Δ of quencing at higher temperatures (35 °C) (Supplementary Figure S4). These results suggested a complex mechanism of action. In order to understand the nature of interaction between Cu(II) and H1 histones, we used a hydrophobic dye such as ANS^36^ to investigate the structural changes of H1 histones Cu(II) induced. In particular, we observed an increase in the ANS fluorescence intensity of 4 and 10 times for somatic and native/deguanidinated derivatives of sperm H1 histones, respectively, with a blue shift of 515, 490 and 480 nm for somatic, deguanidinated and native sperm H1 histones, respectively (Supplementary Figure S5). These differences indicated strong binding between native sperm H1 histones and ANS with respect to somatic H1 histones. Addition of increasing concentrations of CuCl_2_ caused a decrease in the fluorescence intensity (Supplementary Figure S4) for all H1 histones-ANS complexes suggesting that Cu(II) displaces ANS from its binding site, since the decrease of fluorescence intensity is indicative of the transfer of the probe from a non-polar to an aqueous environment.

**Figure 6:**
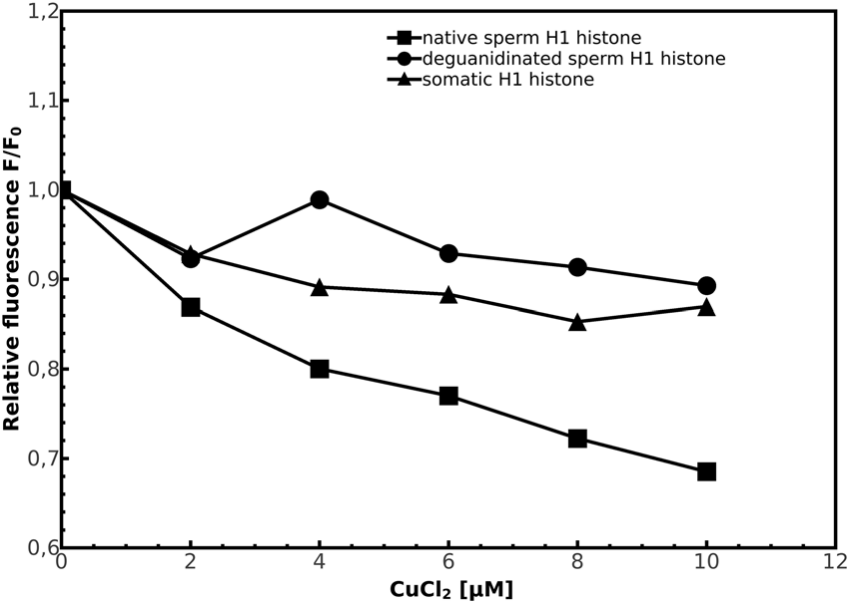
Relative fluorescence plot F/F_0_ of ANS-H1 histones complex in presence of increasing concentrations of CuCl_2_ in the range from 0 to 10 μM.

In Fig. 6, the plot of relative fluorescence intensity of protein-ANS complexes (F/F_0_, where F and F_0_ are the fluorescence intensity of ANS in the presence and absence of CuCl_2_, respectively) versus CuCl_2_ concentration, is shown. The extent of quenching by addition of CuCl_2_ was lower for somatic and deguanidinated derivatives sperm H1 histones as compared to native sperm H1 histones, indicating the presence of an higher amount of polar external binding sites for ANS on the protein surface of native sperm H1 histones.

## Discussion

A number of studies exist on DNA oxidative damage induced by copper and H_2_O_2_^37^. Furthermore, some papers have previously reported DNA oxidative damage induced by histone peptides, in presence of metals and H_2_O_2_ used at least 100 μM^27,38,39^. The common feature of these peptides was the presence of arginine residues and the authors explained an additional site-specific damage at guanine residues of DNA by and a selective binding between arginine and guanine. To date, nobody has investigated in detail the role of arginines in this process, in particular in complex with metals. In fact, it has long been known the possibility of formation of several binary and ternary copper-arginine complexes^40–44^ and it has been reported the involvement of the amidic groups on the backbone of arginine residues in the coordination mode of Cu(II) ion as the case of Cap43 protein fragment T_1_R_2_S_3_R_4_S_5_H_6_T_7_S_8_E_9_G_10_^45^. So, in this work, we investigated the involvement of arginine residues, present in H1 histones, in DNA breakage in presence of low concentrations of CuCl_2_ and H_2_O_2_. To this aim we used sperm and somatic H1 histones from *Ch*.*v* and C.T., respectively. These two types of H1 histones differ substantially in their arginine content (12.6 mol % and 1.8 mol % respectively). First of all we performed EMSA using plasmid pGEM3 DNA and the two types of H1 histones with and without CuCl_2_. The results indicated that CuCl_2_ doesn’t change the DNA binding mode of the two types of H1 histones, previously defined “intermediate mode” for somatic H1 histones and “all or nothing mode” for sperm H1 histones^30^. Somatic H1 histones DNA binding mode was reported also for *Mytilus galloprovincialis* protamine-like proteins PLII and PLIV^46^. Sperm H1 histones DNA binding mode was instead observed also for *Mytilus galloprovincialis* protamine-like proteins PL-III^46^ and for *Chaetopterus variopedatus* protamine-like (*Cv*PL)^47^. In these latter three types of proteins the possibility exists of interactions between ε-amine groups of lysine and guanidino groups of arginines mediated by an intermediate anion^30,48–50^. Such type of interactions, lead to self-association of these proteins and are favored in presence of DNA, where the DNA phosphates represent the intermediate anions^48^.

Although, CuCl_2_ doesn’t change the DNA binding mode, in the case of native sperm H1 histones, determines an increase of DNA binding affinity (Fig. 1b lanes 5 and 9) and promotes DNA breakage, Cu(II)/H_2_O_2_-induced, at low H1/DNA (w/w) ratios (Fig. 2b, lanes 6 and 9). At these H1/DNA ratios it’s possible to evaluate the DNA breakage by the changes of plasmid DNA topological state from supercoiled to relaxed form. Our results are in line with those reported by Zavitsanos *et al*.^38^ that demonstrated that peptides mimicking the H2B histone fold domain (i.e. H2B32-62 and H2B63-93), as well as its N terminal tail (H2B1-31) are able to enhance copper induced single and double strand scission of plasmid DNA^38^. The authors demonstrated also that among all three peptides analyzed, the H2B1-31, arginine rich, seems to cause the highest yield of plasmid relaxation, having the highest affinity for plasmid DNA^38^ as we demonstrated for native sperm H1 histones. The ability to induce DNA breakage, observed in H1 histones, in the presence of Cu(II), can be ascribed to structural changes, but the CD analysis highlighted that the general organization of the secondary structure of sperm H1 histone remains unchanged (Supplementary Figure S2), supporting the hypothesis that the observed effects Cu(II) induced on the functional/structural behavior of the proteins should be due to tertiary/quaternary structure interactions. Changes in intrinsic fluorescence of a protein, such as fluorescence quenching, is a sensitive tool to obtain information about protein-ligand interactions. So we exploited the fluorescence quenching of tyrosine residues present in the sperm H1 histone, as determined by amino acid composition^51^, to obtain information on the type of interactions between copper and the protein surface. In fact, fluorescence quenching could be classified as dynamic or static depending on if caused by collisional encounters between the fluorophore and the quencher, or resulted from the formation of stable compound between fluorophore and quencher, respectively^52^. However, measuring the dependence of the Stern-Volmer constant (K_SV_) on temperature, we obtained non-linear Stern-Volmer plots at 25 °C and the absence of quenching at 35 °C, indicative of complex interactions^52,53^. As a matter of fact a specific interaction between Cu(II) and H1 histones, was supported by their PK resistance in presence of CuCl_2_ and not in presence of NiCl_2_ (Fig. 3d lanes 10 and 12). We exclude also a possible inhibitory effect of CuCl_2_ on the PK activity, since the copper-induced inhibition of PK activity was reported only at concentrations higher than 200 μM^54,55^. PK resistence observed on H1 histones could be ascribed just to a protein conformational change, copper-induced, in this molecule as reported for ovine prion protein^34^. Moreover, native sperm H1 histones result highly resistant to PK digestion even after an overnight treatment in presence of CuCl_2_, differently from somatic H1 histones which contain a low amount of arginine residues.

In order to analyze the possible involvement of arginines in the different PK resistance of somatic and sperm H1 histones, we have chemically modified arginine in ornithine residues in sperm H1 histones. The deguanidinated sperm H1 histone derivatives (K/R = 14) resulted more susceptible to PK digestion than native molecule (K/R = 2) in a manner more similar to somatic H1 histones (K/R = 15), indicating that the conversion of arginine to ornithine residues causes a decrease in PK resistance. These results indicate the relevance of K/R ratio in these H1 histones properties because the deguanidinated sperm H1 histone derivatives present PK resistance and K/R ratio similar to somatic H1 histones. Unfortunately, the low content in aromatic residues of H1 histones make difficult to use intrinsic fluorescence for structural studies of these proteins. An alternative to the use of intrinsic fluorescence for measuring changes on protein surface was the use of external probes, such as ANS. The increase of fluorescence intensity and blue shift of fluorescence emission maxima observed for ANS are generally attributed to the hydrophobicity of binding sites and the restricted mobility of ANS^36^. However, it has been reported that ANS binds also to polar external sites of proteins, which are exposed to the aqueous phase^56^, in particular strongly binds arginine and lysine residues of proteins through ion pair formation, although, the contribution of ANS fluorescence from these external binding sites is much less compared to that from buried sites.

In our experiments, we observed a decrease of blue shift of ANS fluorescence emission maxima in sperm H1 histone deguanidinated derivatives with respect to native molecules that could be explained by the lesser number of arginine residues that bind ANS, following the conversion of arginine in ornithine residues.

We measured a decrease in the fluorescence intensity of H1 histones-ANS complexes after addition of CuCl_2_, indicating a competition from the same binding sites on the protein surface between Cu(II) and the fluorescent probe. The percentage quenching [(F_0_ − F)/F_0_ × 100] of the native sperm H1 histones-ANS complex in presence of 10 μM CuCl_2_ was 31,5%, about 3 times higher respect to the percentage quenching observed for deguanidinated sperm H1 histone derivatives (10,7%) and somatic H1 histones (12,9%). These observations strongly support the involvement of arginine residues on the protein surface in H1 histones-Cu(II) interactions.

**Figure 7:**
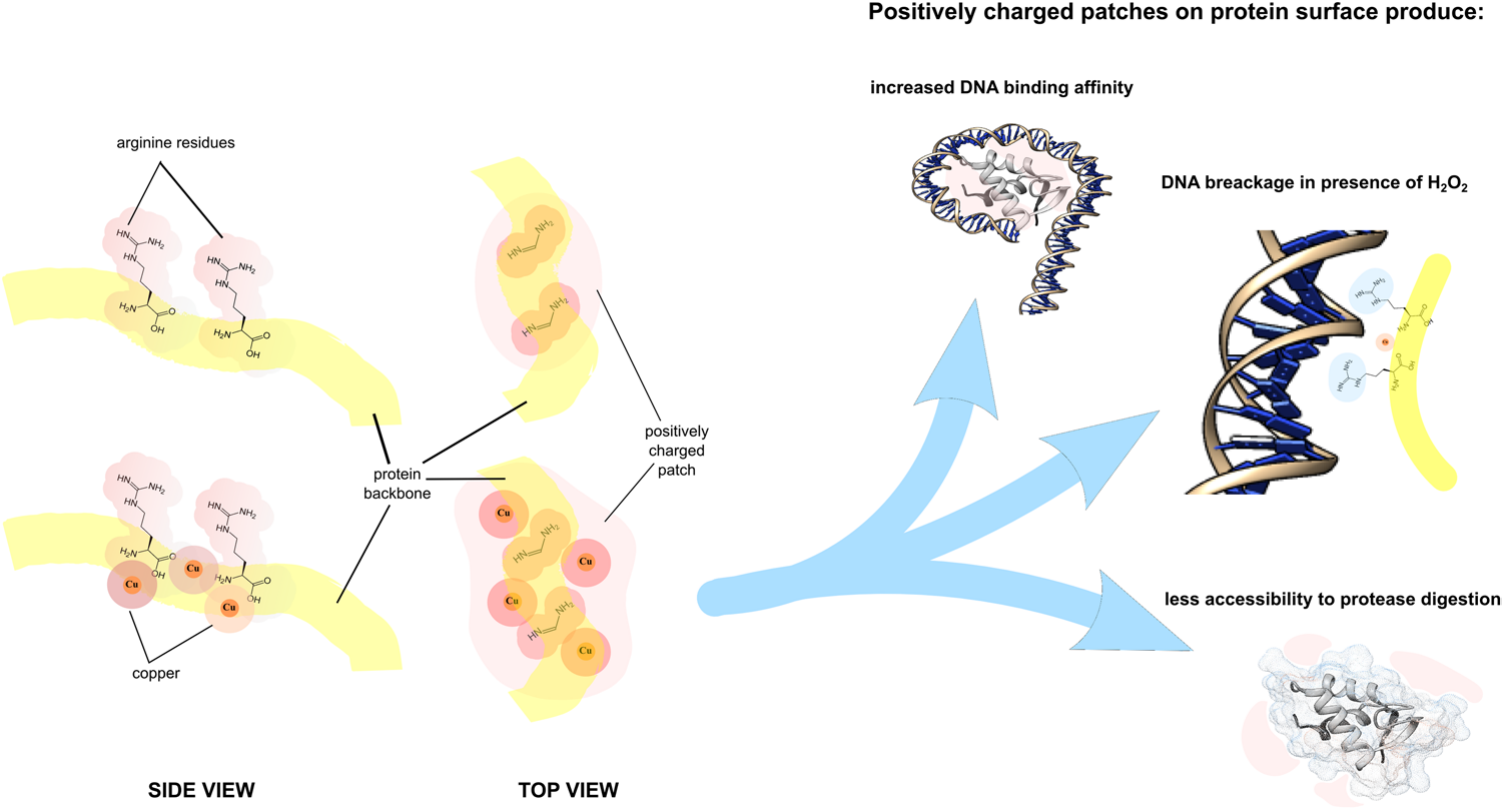
Model of interaction between Cu(II) and the amidic groups on the backbone of arginine residues on protein surface of sperm H1 histones.

All the data obtained in this work permit to hypothesize a model of interaction between Cu(II) and backbone of arginine residues, since arginine guanidinium groups are not potential donors for Cu(II), on protein surface of sperm H1 histones (Fig. 7). Evidences that amidic groups in the backbone of peptides and proteins are involved in the coordination mode of Cu(II)^45^, support the hypothesis that Cu(II) interacts with negatively charged groups on the protein backbone, such as the amidic groups on the backbone of arginine residues, favored by a reorganization of the side-chains of arginine residues metal-induced^29,45^. This arrangement generates more wide positively charged patches on protein surface, because of the inclusion of charged Cu(II) ions in the structure, that could produce several effects. First of all an increase in DNA binding surface, affecting the strength of interactions between the negatively charged phosphate groups and the positive arginine-Cu(II) patches, that in turn results in a higher DNA binding affinity of sperm H1 histones. Consequently, the availability of Cu(II) ions near the binding surface between DNA and native sperm H1 histones, make possible that the addition of H_2_O_2_ to this complex promotes the Fenton reaction in DNA proximity, determining its breakage. Moreover, these patches could represent a sort of barrier to protease accessibility to its target residues, because of the increase of repulsive forces for the addition of positive charges and a probable steric hindrance due to the side-chains reorganization. This could explain the higher PK digestion resistance of sperm H1 histones in presence of copper. In conclusion, these observations support the existence of Cu(II) effects arginine-dependent, that provides new insight in copper toxicity mechanisms. Further, although histones are known to protect DNA, in particular conditions, arginine-rich histones appear to participate in copper-dependent oxidative damage of DNA. Of course, the precise mechanisms by which arginine residues, interacting with copper ions, can induce a higher PK resistance in native sperm H1 histones and make they able of inducing oxidative DNA damage will have to be elucidated by further studies.

## Materials and Methods

### Materials

Chemical reagents and somatic H1 histone from C.T. were obtained from Sigma (USA).

### Preparation of *Ch*.*v*. histone H1

*Ch*.*v*. sperm H1 histone was purified as described in De Petrocellis *et al*.^51^. The protein concentration was determined on the basis of the known tyrosine content: one tyrosine in C.T. somatic H1 molecule^57^ and two tyrosines in *Ch*.*v*. sperm H1 molecule^51^ using ε276 = 1340 cm^−1^ M^−1^ according to Giancotti *et al*.^58^.

### Amino-acid side chain modifications

Deguanidination reaction on *Ch*.*v*. sperm H1 histone was performed as described in Piscopo *et al*.^30^. Unreacted amino groups were titrated with 2,4,6 trinitro-benzene-sulfonic acid^59^.

### Acetic acid–urea polyacrylamide gel electrophoresis (AU-PAGE)

Native and modified *Ch*. *v*. sperm H1 histones and C.T. H1 histones were analyzed by AU-PAGE as described in Vassalli *et al*.^46^.

### PK digestions on H1 histones

Digestion of H1 histones with PK (Promega) was carried out as followed: 100 µg of H1 histone were resuspended in 500 µL of 10 mM Tris HCl pH 8 and digested at 37 °C with 5 µg of PK in presence of 10 µM CuCl_2_ in the range from 5′ to overnight. The products of reaction were analyzed by AU-PAGE using the H1 histones not digested as control. All experiments were performed at least three times.

### Far-UV CD spectroscopy

The circular dichroism (CD) analyses was carried out using a Jasco spectropolarimeter model J-810, which was equipped with a Julabo F25-ME temperature controller (Julabo GmbH) and calibrated with a standard solution of (+)-10-camphorsulfonic acid. CD measurement in the far-UV was performed in a 0.1 cm optical path length cuvette (STARNA), using a protein concentration of 0.1 mg/mL in 10 mM TRIS-HCl pH 8.0 buffer. CD spectra were acquired in presence of increasing concentrations of CuCl_2_ in the range from 0 to 10 mM. Photomultiplier absorbance did not exceed 600 V in the spectral regions measured. Each spectrum was signal averaged at least three times and smoothed with CD software Spectra Manager Ver. 1.53 (Jasco Corporation). All measurements were performed at least three times at room temperatures under a nitrogen flow of 3 L/h.

### Fluorescence spectroscopy

The fluorescence analyses was carried out in a 1 cm optical path length cuvette (STARNA) using a Jasco spectrofluorimeter model FP 8200, equipped with a Julabo F25-HD temperature controller (Julabo GmbH). Intrinsic fluorescence measurements were performed measuring the fluorescence emission of Tryptophan residues in the range from 310 to 460 nm after excitation at 290 nm. Measure of the fluorescence quenching has been carried out on sperm H1 histones at the concentrations of 0,10 mg/mL in 10 mM TRIS-HCl pH 8.0 buffer, at 15, 25 and 35 °C. Measurement in presence of ANS (5 μ concentration) was performed measuring the fluorescence emission in the range from 420 to 600 nm after excitation at 350 nm, in presence of a protein concentration of 0.10 mg/mL in 10 mM TRIS-HCl pH 8.0 buffer. Spectra of intrinsic and extrinsic fluorescence were acquired in presence of increasing concentrations of CuCl_2_ in the range from 0 to 10 μM. Photomultiplier absorbance did not exceed 600 V in the spectral regions measured. Each spectrum was signal averaged at least three times and smoothed with the software Spectra Manager Ver. 2.09 (Jasco Corporation). All measurements were performed at least three times at room temperatures.

### Preparation of DNA

The DNA used in all experiments was pGEM3 DNA (2867 bp) prepared from *Escherichia coli* HB 101 cells transformed by the plasmid. Plasmid pGEM3 was purified using the method described in Carbone *et al*.^60^ and analyzed by gel electrophoresis on 1% agarose gels in 89 mM Tris-HC1 pH 8.0, 2 mM EDTA and 89 mM boric acid (TBE).

### Analysis of the effect of CuCl_2_ on DNA binding of sperm and somatic H1 histones by EMSA

The effect of sperm and somatic H1 histones on DNA was analyzed by Electrophoretic Mobility Shift Assay (EMSA). Protein to DNA (w/w) ratios were between 0 and 3 as indicated in each experiment. The reported amounts (see results) of H1 histones were added to 250 ng of circular pGEM3 DNA in a final volume of 27 µL. In the samples containing 10 µM CuCl_2,_ H1 histones were incubated for 10 min with CuCl_2_, at room temperature before incubation with DNA which was for 10 min at room temperature. At the end of incubation, just before electrophoresis analysis, all samples were added with 3 µL of TBE 10X (in order to obtain TBE 1X final concentration) and analyzed on 1% agarose gel in TBE 1X final concentration. TBE was added to the samples just before running the gels in order to avoid EDTA coordination of Cu ions. Electrophoresis was carried out at 100 V for 30 minutes. DNA migration was visualized by staining gels with ethidium bromide (2 mg/mL) after electrophoresis. All experiments were performed at least three times.

### Analysis of Cu(II)/H_2_O_2_-induced DNA breakage in presence of H1 histones

pGEM3 plasmid DNA breakage in presence of sperm and somatic H1 histones, 10 μM CuCl_2_ and 10 μM hydrogen peroxide (H_2_O_2_), was analyzed on 1% agarose gel in TBE 1X final concentration. The preparation of samples followed the same modality described in the previous paragraph with the only difference that protein to DNA (w/w) ratios were 0.1 and 0.2. H_2_O_2_ was the last component to be added and the samples were incubated for 30 minutes at 37 °C. At the end of incubation, samples were added with 3 µL of TBE 10X (in order to obtain TBE 1X final concentration) just before electrophoresis analysis in order to avoid EDTA coordination of Cu ions. Samples containing DNA alone; DNA + CuCl_2_; DNA + CuCl_2_ and H_2_O_2_ were used as control. Electrophoresis was carried out at 100 V for 30 minutes. DNA migration was visualized by staining agarose gels with ethidium bromide (2 µg/mL) after electrophoresis. All experiments were performed at least three times.

### Data availability

No datasets were generated or analysed during the current study.

## Electronic supplementary material

Supplementary Figures
