# Supplementary material for: Relevance of arginine residues in Cu(II)-induced DNA breakage and Proteinase K resistance of H1 histones
Source: Sci Rep. 2018 May 9;8:7414. doi: 10.1038/s41598-018-25784-z (PMC5943286; doi:10.1038/s41598-018-25784-z)
Supplement: Supplementary file 1 — Supplementary Figures [file 41598_2018_25784_MOESM1_ESM.pdf]

Relevance of arginine residues in Cu(II)-induced DNA breakage and Proteinase K resistance of H1 histones

Marina Piscopo[1*], Marco Trifuoggi[2], Carmela Scarano[1], Carla Gori[3], Antonella Giarra[2] & Ferdinando Febbraio[3*]

[1]Dipartimento di Biologia, Università degli Studi di Napoli Federico II, 80126, Napoli, Italy
[2]Dipartimento di Scienze Chimiche, Università degli Studi di Napoli Federico II, Napoli, Italy
[3]CNR, Institute of Protein Biochemistry, 80131, Napoli, Italy.

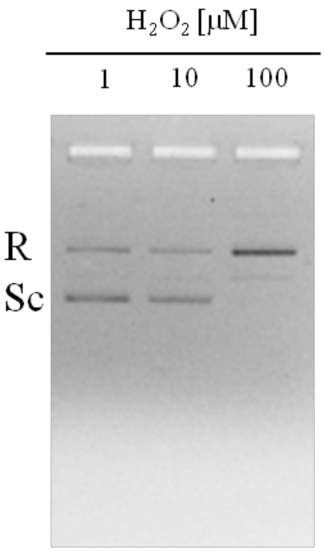

Figure S1. Evaluation of pGEM3 DNA plasmid breakage in presence of H_2O_2 concentrations. DNA breakage is evaluated by the conversion of supercoiled to relaxed form of 250 ng of circular pGEM3 DNA plasmid in presence of increasing concentration of H_2O_2 (1, 10 and 100 µM). pGEM3 DNA plasmid breakage, evaluated by the conversion of supercoiled to relaxed plasmid DNA form is observed only in the samples treated with H_2O_2 100 µM.

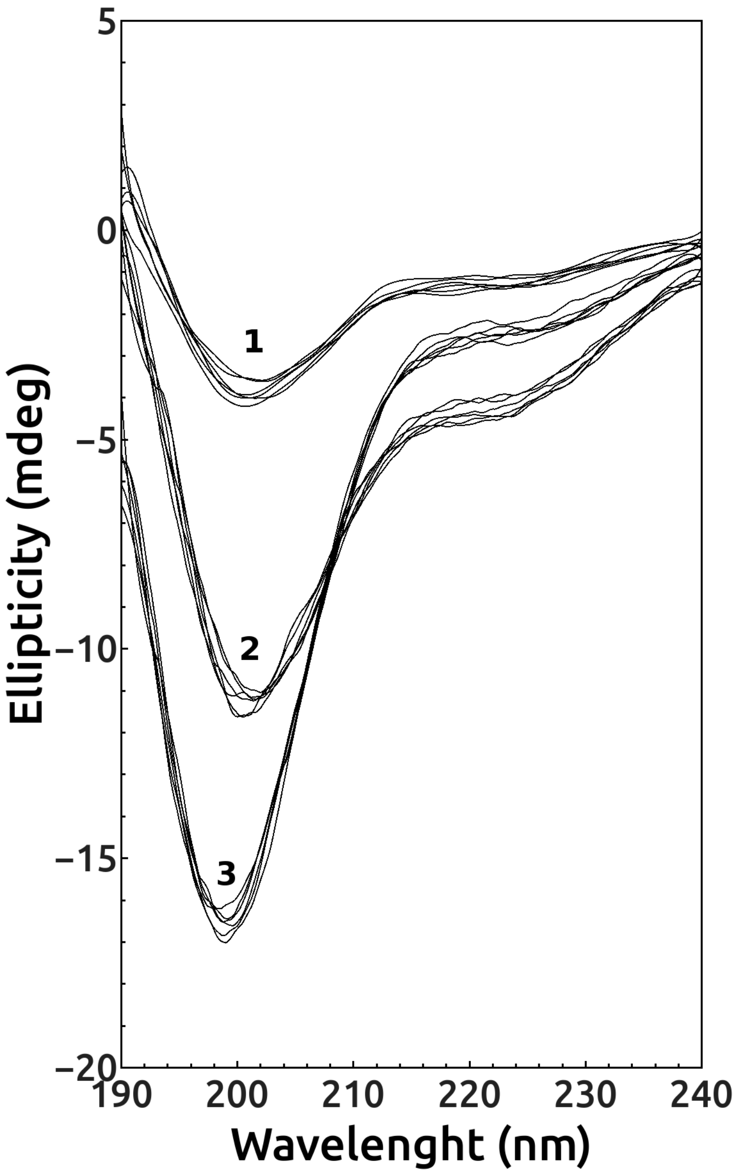

Figure S2. Far-UV CD spectra of H1 histones in presence of increasing $CuCl_2$ concentrations. Plot of far-UV CD spectra of sperm histones (2), its deguanidinated derivatives (1) and somatic H1 histones (3) in presence of increasing concentrations of $CuCl_2$ in the range from 0 to 10 μM.

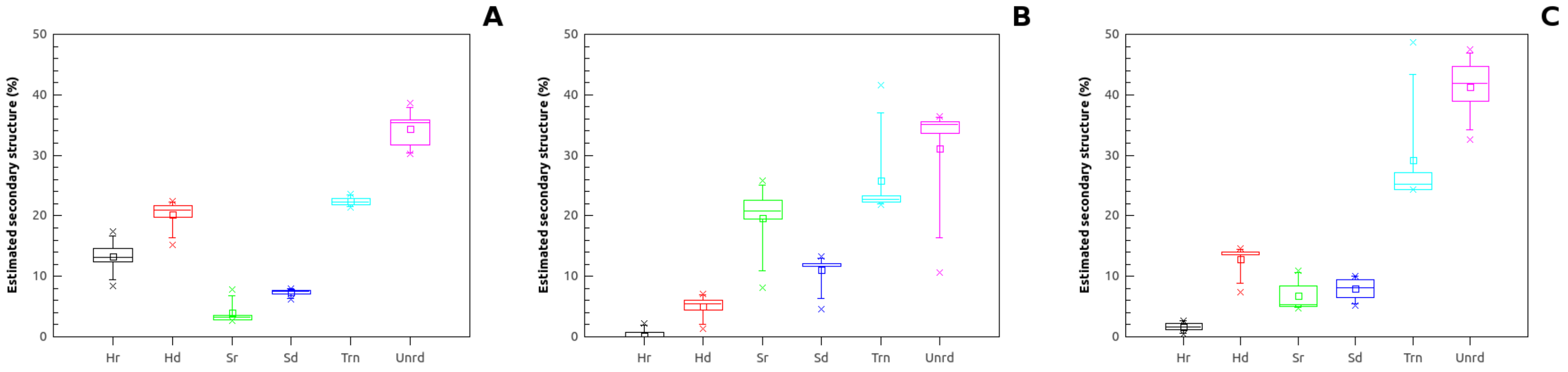

Figure S3. Plot of changes in secondary structure at increasing $CuCl_2$ concentrations. Box-plot representation of changes in secondary structure estimation at increasing $CuCl_2$ concentrations of A) sperm H1 histones, B) deguanidinated derivatives H1 histones and C) somatic H1 histones. The lines inside the boxes represent the medians. The boxes represent the first (Q1) and the third (Q3) quartiles, and the two whiskers represent the minimum and the maximum values, except for outliers. Cross represent outliers. In black and red the α-helices regular (Hr) and distorted (Hd) fractions, respectively, in green and blue the β-sheets regular (Sr) and distorted (Sd) fractions, respectively, in light blue the β-turn (Trn) fraction and in pink the unordered (Unrd) fraction.

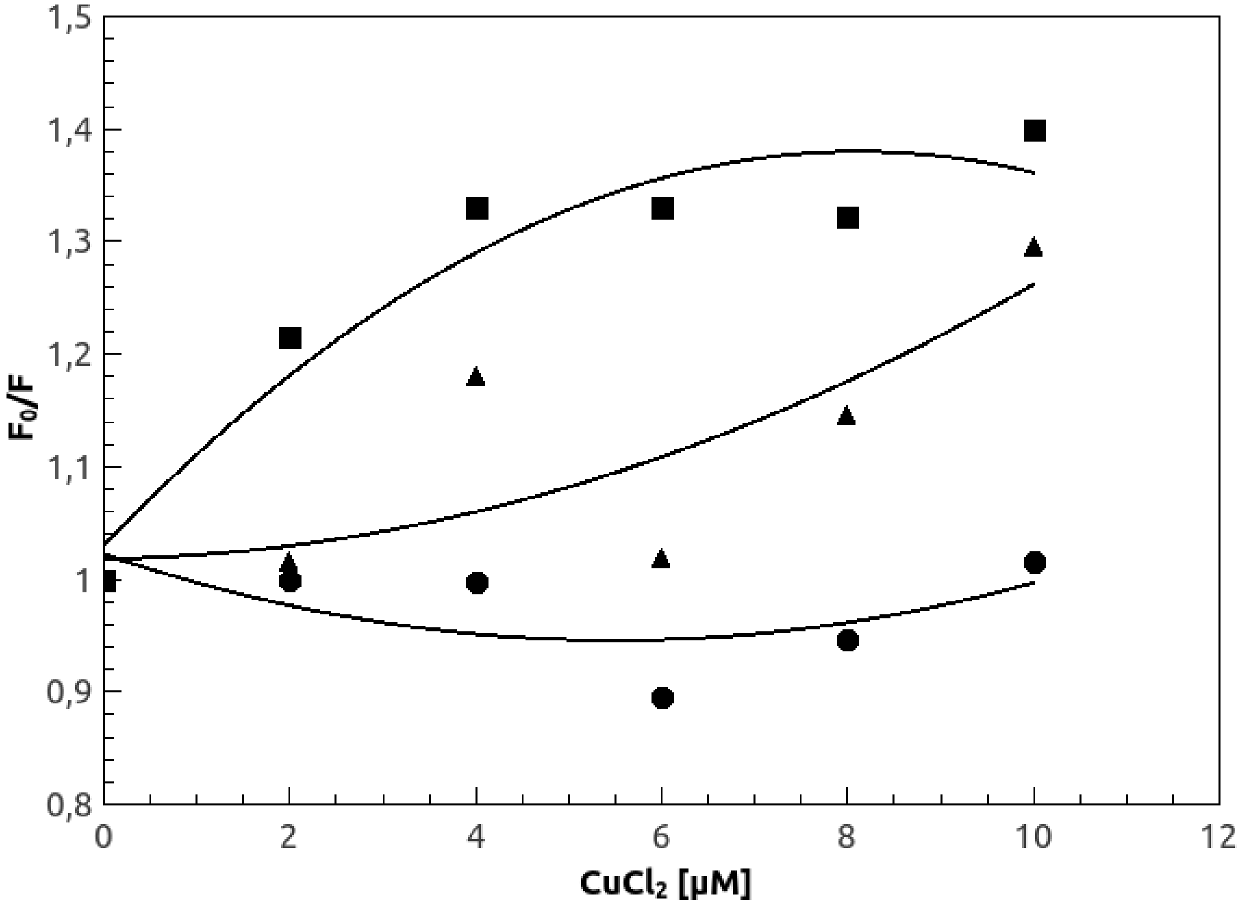

Figure S4. Stern-Volmer plot at different temperature of sperm H1 histones fluorescence. Stern-Volmer plot of F_0/F against increasing $CuCl_2$ concentrations in the range from 0 to 10 µM of sperm H1 histones intrinsic fluorescence at 15 (triangle), 25 (square) and 35 °C (circle).

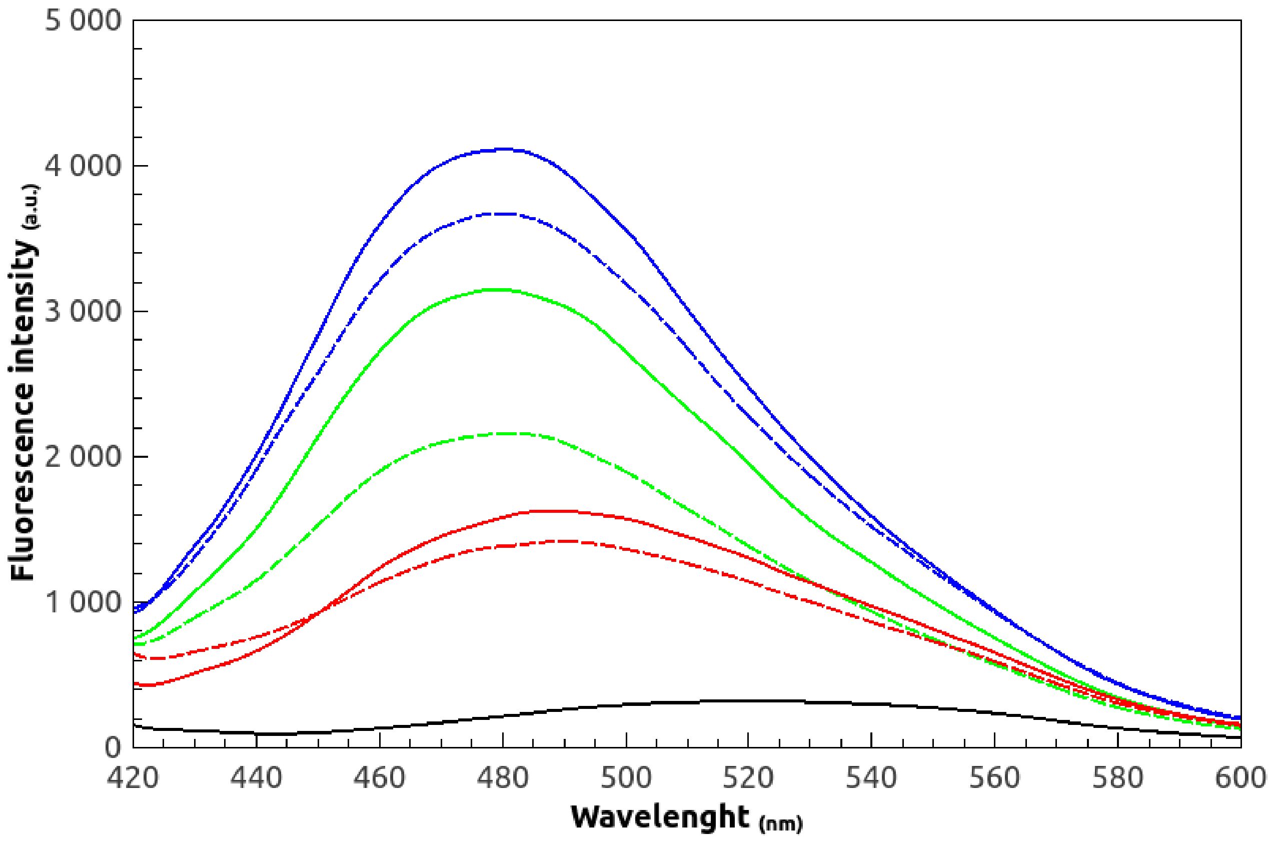

Figure S5. Fluorescence spectra of H1 histones in presence of ANS. Fluorescence spectra at the Excitation wavelength of 350 nm of sperm (green), deguanidinated derivatives (blue) and somatic (red) H1 histones in presence of ANS 5 µM (continued line) and after addition of 10 µM $CuCl_2$ (dashed line). Reference spectra of ANS is in black.
